# Measurement of Ultra-Low Frequency Vibrations Using an Atom Interferometer

**DOI:** 10.3390/s25134136

**Published:** 2025-07-02

**Authors:** Zenghan Ma, Wei Zhuang, Yang Zhao, Chuanjing Ruan, Qin Tian, Jiamin Yao, Jinyang Feng, Shuqing Wu, Fang Fang, Ling Wan

**Affiliations:** 1 College of Instrumentation and Electrical Engineering, Jilin University, Changchun 130026, China; mazh21@mails.jlu.edu.cn; 2 National Institute of Metrology, Beijing 100029, China; zhaoyang@nim.ac.cn (Y.Z.); 21111076@bjtu.edu.cn (C.R.); 22110072@bjtu.edu.cn (Q.T.); yaojm@nim.ac.cn (J.Y.); fengjy@nim.ac.cn (J.F.); wushq@nim.ac.cn (S.W.); fangf@nim.ac.cn (F.F.); 3 Key Laboratory of State Administration for Market Regulation (Time Frequency and Gravity Primary Standard), Beijing 100029, China; 4 School of Automation and Intelligence, Beijing Jiaotong University, Beijing 100044, China

**Keywords:** atom interferometer, low-frequency vibration measurement, vibration isolation

## Abstract

Measuring low-frequency and ultra-low-frequency vibration signals is of critical importance in fields such as structural mechanics, geological exploration, aerospace, precision machining, and biomedicine. Existing methods face limitations in achieving both ultra-low-frequency range and high precision. We present an ultra-low-frequency vibration measurement method based on the atom interferometer, capable of measuring vibration signals from 0.01 Hz to DC. The performance of measurement was experimentally demonstrated for vibrations between 0.007 Hz and 0.01 Hz, achieving a sensitivity of $1.1\;\mu m/s^{2}/\sqrt{\mathrm{Hz}}$. Incorporating active vibration isolation can further enhance the measurement range, increasing the optimal sensitivity to $0.2\;\mu m/s^{2}/\sqrt{\mathrm{Hz}}$.

## 1. Introduction

Owing to the low frequency, high amplitude, and destructive potential of low-frequency and ultra-low-frequency vibrations, it is essential to detect these vibrations in fields such as structural mechanics [1], geological exploration [2], aerospace engineering [3], precision manufacturing [4], and biomedical sciences [5]. Currently, the principal methodologies for detecting such vibrations encompass accelerometers, seismometers, and laser vibrometers. Table 1 presents a comparison of the performance of various schemes for measuring ultra-low-frequency vibrations. Accelerometers typically demonstrate low signal-to-noise ratios (SNRs) for signals below 1 Hz, while simultaneously requiring advanced calibration techniques [1,6]. Seismometers, while capable of detecting vibrations as low as 0.0071 Hz, require complex calibration and signal processing to convert their voltage outputs into acceleration values. In contrast, the present method is engineered to directly measure true vibration acceleration, offering distinct advantages in physical relevance [2,7]. Laser vibrometers can measure vibration signals down to direct current (DC). A comparative analysis of various ultra-low-frequency vibration measurement techniques has been conducted, as presented in Table 1. However, their resolution is limited for ultra-low-frequency vibrations [8,9]. Therefore, due to the limitations of existing methods, there is a growing need for more effective solutions to detect the impact of low frequency and ultra low frequency vibrations in various applications.

The atom interferometer is an interferometer that relies on the superposition of atomic quantum states, measuring the phase difference of the atom interference based on the wave nature of atoms [10,11]. Leveraging the remarkable development of laser cooling technology, the atom interferometer has made breakthrough advancements in acceleration measurement [12], gyroscopes [13,14], and gravity measurement [15,16,17,18]. During these measurements, low-frequency vibrations significantly impact measurement noise [19,20]. Therefore, many vibration reduction techniques have been introduced, including the vibration isolation system [21,22,23,24,25,26] and vibration correction [27,28,29]. In this work, we introduce an alternative approach to low-frequency vibration detection using atom interferometry. Compared to seismometers requiring frequent calibration, atom interferometry enables absolute vibration measurements from DC upwards without the need for sensor calibration, offering inherent long-term stability. Compared with laser vibrometers, this approach possesses exceptional sensitivity, long-term stability, and accuracy. Additionally, the theoretical lower limit of frequency measurement by the atom interferometer theoretically extends down to 0 Hz. Furthermore, atom interferometers do not require a stationary platform. Therefore, the atom interferometer represents an excellent candidate for the measurement of low-frequency vibrations.

**Table 1:** Methods and performances for measuring ultra-low frequency vibrations.

	Frequency Ranges	Self-Noise
accelerometers [30]	DC—430 Hz	$3.1\times 10^{-7}\;m/s^{2}/\sqrt{\mathrm{Hz}}$ (0.01 Hz)
seismometers [31]	0.0083 Hz–80 Hz	$1\times 10^{-9}\;m/s^{2}/\sqrt{\mathrm{Hz}}$ (0.01 Hz)
laser vibrometers [32]	DC—22 kHz	$2\times 10^{-8}\;m/s/\sqrt{\mathrm{Hz}}$ (0.5 Hz)
atom interferometer	DC—100 Hz	$2\times 10^{-7}\;m/s^{2}/\sqrt{\mathrm{Hz}}$ (0.01 Hz this article) $1\times 10^{-8}\;m/s^{2}/\sqrt{\mathrm{Hz}}$ (0.01 Hz future) [33]

In this paper, we introduced the principle of low-frequency vibrations measurement based on atom interferometers and verified its measurement frequency range and sensitivity. Finally, using active vibration isolation, we demonstrated an order of magnitude improvement in measurement sensitivity.

## 2. Principle

**Figure 1:**
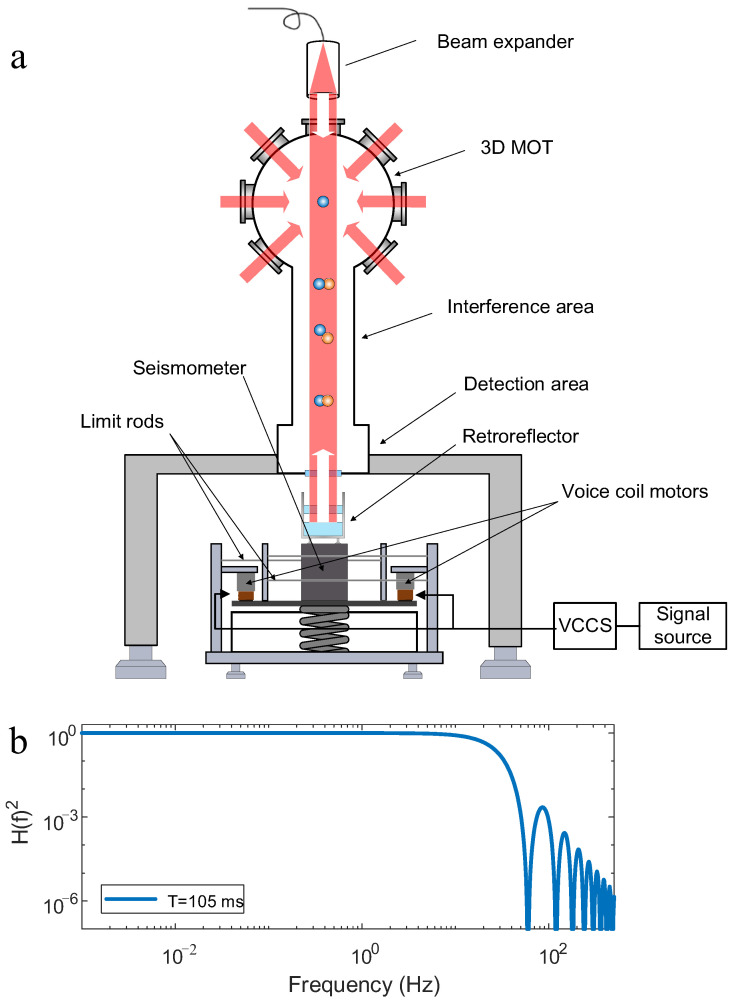
(**a**) The schematic diagram of the atom interferometer. Rb are cooled, and their states are prepared in the MOT zone. Afterward, they are released for atom interferometry, followed by fluorescence detection in the detection zone. The lower section features a device for applying low-frequency vibrations, which can also function as active vibration isolation. (**b**) Atom interferometer transfer function at T = 105 ms.

The working principle and apparatus of our atom interferometer have been described in detail elsewhere [18]. Here we provide a brief description as shown in Figure 1a. Initially, Rb87 atoms are cooled and trapped using six laser beams in a three-dimensional magneto-optical trap (3D-MOT). The atomic cloud is then further cooled to 2 µK in an optical molasses. Next, the atoms are prepared in the state $\left|F=1,\;m_{F}=0\right\rangle$ by applying microwave and Raman optical selection. Subsequently, the atoms are released and enter a free-fall phase within the interference region, where a sequence of π/2-π-π/2 Raman pulses constructs a Mach–Zehnder-type atom interferometer. Finally, the atomic population is measured via fluorescence detection in the designated detection zone. Using normalized detection methods, the population proportion *P* of atoms in the hyperfine state *F* = 2 is derived, expressed as:

(1) $$P=P_{m}-\frac{C}{2}\cos\Delta \Phi_{m}=P_{m}-\frac{C}{2}\cos\left(\Delta \Phi_{g}+\Delta \varphi_{\mathrm{vib}}+\Delta \varphi_{\mathrm{others}}\right),$$

where $P_{m}$ is the mean proportion of atoms in the state *F* = 2, and C is the contrast. The actual phase shift $\Delta \Phi_{m}$ can be obtained by summing the phase shift $\Delta \Phi_{g}$ due to gravity, the phase shift due to vibrations $\Delta \varphi_{\mathrm{vib}}$, and the phase shift due to other noise sources $\Delta \varphi_{\mathrm{others}}$. The phase shift due to vibrations $\Delta \varphi_{\mathrm{vib}}$ can be calculated using the following formula [2,3]:

(2) $$\Delta \varphi_{\mathrm{vib}}=k_{eff}\int_{0}^{2T}S\left(t\right)v_{m}\left(t\right)dt,$$

where $k_{eff}$ is the effective wave vector of the Raman laser beams, T is the time interval between two successive Raman laser pulses, and $v_{m}(t)$ is the vertical vibration velocity of the retroreflector. Neglecting the duration of the Raman pulses, the sensitivity function S(t) can be approximated as [34]:

(3) $$S\left(t\right)=\left\{\begin{array}{c} -1\;t_{start}<t<t_{start}+T\\ 1\;t_{start}+T<t<t_{start}+2T\; \end{array}\right.,$$

where $t_{start}$ represents the starting time of each measurement cycle. We suppose a vibration signal $a_{in}(t)$ applied to the retroreflector with the expression:

(4) $$a_{in}\left(t\right)=A\omega cos\left(\omega t+\varphi \right),$$

where A represents the amplitude, ω represents the angular frequency, and φ  represents the initial phase. Its corresponding acceleration frequency is ω, and amplitude is Aω. By substituting this into (2), the corresponding phase shift due to the vibration can be derived as follows:

(5) $$\Delta \varphi_{\mathrm{vib}}=\frac{{2k}_{eff}A}{\omega }cos\left(\omega nT\_cyc+\omega T+\varphi \right)\left(1-cos\left(\omega T\right)\right),$$

where n is the number of measurement cycles, and T_cyc represents the duration of a single measurement cycle. Under the experimental conditions of this study, T_cyc is set to 1 s. The measurement of vibration acceleration by the atom interferometer $a_{AI}(t)$ is deduced as:

(6) $$a_{AI}\left(n*T\_cyc+T\right)=\Delta \varphi_{\mathrm{vib}}/\left(k_{eff}T^{2}\right)=\frac{4{sin}^{2}\left(\omega T/2\right)}{{\left(\omega T\right)}^{2}}A\omega cos\left(\omega n+\omega T+\varphi \right),$$

The corresponding amplitude transfer function $\left|H\right|$ represents the system’s response to varying frequency components of the input signal. In this analysis, the influence of vibration during the Raman pulse duration is neglected. This is because the Raman pulse duration in our experiment is extremely short (25 μs), and the effect of vibration during the Raman pulse duration on the amplitude transfer function becomes significant only at frequencies above 40 kHz. The amplitude transfer function is expressed as:

(7) $$\left|H\right|=\frac{a_{AI}(n+T)}{a_{in}(n+T)}=\frac{4{sin}^{2}\left(\omega T/2\right)}{{\left(\omega T\right)}^{2}}.$$

Figure 1b shows the transfer function with *T* set to 105 ms. The transfer function indicates that the atom interferometer exhibits high sensitivity to low-frequency vibrations. At a sampling rate of 1 Hz, the maximum detectable vibration frequency is 0.5 Hz. Substituting 0.5 Hz into (7) yields a vibration attenuation of 0.5‰ at this frequency. Consequently, frequencies below 0.5 Hz exhibit negligible attenuation for practical analysis. For vibrations above 0.5 Hz, the attenuation at 40 Hz is −15 dB. The portion of the power spectral density between 0.5 Hz and 40 Hz is aliased into the 0 to 0.5 Hz range when the sampling rate is 1 Hz. Another key parameter influencing the range and accuracy of this vibration measurement method is T. The vibration measurement process must operate within the linear regime of the interference fringe ($\Delta \varphi_{\mathrm{vib}}$ from 0 to π/2) pattern. Within this linear regime, the measurable acceleration range is inversely proportional to $T^{2}$. Conversely, the sensitivity of measurement improves at the same rate as $T^{2}$ increases. To maximize sensitivity, the interaction time T must be set to its maximum feasible value. In this work, the physical constraints of the experimental apparatus limit $T_{max}$ to 105 ms. With T set to 105 ms, the maximum range for measuring vibration acceleration was 167.6 μm/s^2^. Additional noise sources distinct from vibration noise also contribute to measurement uncertainty. It consists primarily of detection noise, quantum projection noise, and laser phase noise. These noises can further degrade measurement accuracy. Under laboratory conditions, the combined noise floor from other sources is approximately $0.025\;\mu m/s^{2}/\sqrt{\mathrm{Hz}}$. The sum of these contributions is nearly an order of magnitude lower than the total measurement noise floor, so they can be neglected in the current model [18]. Variations in the local gravitational acceleration (g) also introduce errors in vibration sensing. Short-term variations in g remain minimal, with fluctuations below $0.1\;\mu m/s^{2}/\sqrt{\mathrm{Hz}}$ over a 10-min interval. By subtracting the constant g value, its influence can be effectively removed. However, for long-term measurements, the gravitational variations caused by tidal effects must be considered to ensure the accuracy of the vibration measurements.

## 3. Vibration Measurement

### 3.1. Simulation

**Figure 2:**
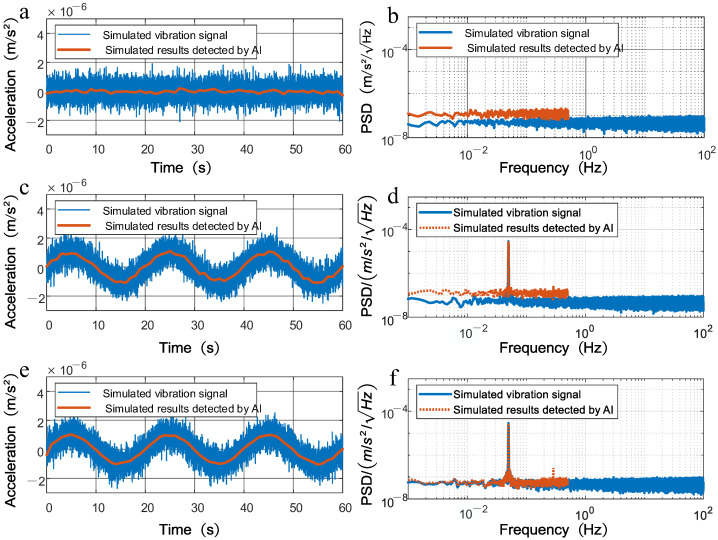
Time-domain plots and PSDs of the input signals and results obtained by simulating the atom interferometer (AI) process. Each row corresponds to a set of simulation experiments. (**a**,**b**) depicts Gaussian white noise with a standard deviation of 0.05 μm/s^2^. (**c**,**d**) adds a sinusoidal vibration signal (amplitude: 1 μm/s^2^, frequency: 0.02 Hz) to the Gaussian white noise. (**e**,**f**) applies a low-pass filter (0.5 Hz to 40 Hz) to the input signal before simulation. Figure (**a**,**c**,**e**) displays time-domain plots of the input and simulation result. Figure (**b**,**d**,**f**) presents the PSD. The red curve indicates the input signal, while the blue curve shows the simulation result.

In the following, we conduct three simulation tests, sampled at 200 Hz. Figure 2a displays the background vibration simulation as the input signal (blue curve), modeled as Gaussian white noise with a standard deviation of 0.05 µm/s^2^. The simulated signal (red curve) is generated by modeling the atom interferometer’s response to the input signal. Since the sampling rate of the atom interferometer is set to 1 Hz, the time-domain plot in Figure 2a demonstrates a low-pass filter effect. Figure 2b shows the PSD of both the input and simulated signals. The PSD amplitude of the simulation result is 1.5 times larger than that of the input signal. This occurs due to the aliasing effect, which stacks the spectral density from 0.5 Hz to 40 Hz into the region below 0.5 Hz. Subsequently, a sinusoidal vibration signal with an amplitude of 1 $\mu m/s^{2}$ and a frequency of 0.02 Hz was superimposed onto the background vibration to form the new input signal. Figure 2c shows the time-domain plot, where the sinusoidal vibration signal is clearly visible. This method effectively suppresses high-frequency vibrations. The PSD in Figure 2d shows that the simulated signal’s amplitude at 0.02 Hz coincides with the input signal’s amplitude at the same frequency. The Gaussian white noise component of the simulation result is still 1.5 times larger than that of the input signal like Figure 2b. To characterize aliasing effects and improve resolution, a band-stop filter (0.5–40 Hz) was applied to the input signal, yielding a filtered input signal for modeling a new simulated signal. Figure 2e illustrates a smoother amplitude in the simulated signal. Figure 2f shows that the PSD of the simulated signal aligns closely with the filtered input signal below 0.5 Hz. The filtered input signal results in approximately a 2% difference in the simulated signal. The background vibration observed in the simulated signal is of the same order of magnitude as the input signal. Notably, the PSD of the input signal at 0.02Hz is on the order of $10^{-5}\;m/s^{2}/\sqrt{\mathrm{Hz}}$, while the aliasing effects (on the order of $10^{-7}\;m/s^{2}/\sqrt{\mathrm{Hz}}$) are far smaller than the PSD of the input signal at 0.02 Hz, rendering them negligible.

### 3.2. Experiments

We designed a vibration source system, as shown in Figure 1a, consisting of a commercial passive isolator with horizontal constraints, voice coil motors, a voltage-controlled current source (VCCS), and a signal source. The commercial passive isolator selected for the experimental setup is the Minus K 25-BM10 (Minus K Technology, Los Angeles, CA, USA). Its vertical frequency is tunable to 0.5 Hz across the full payload range of 4.5–14 kg. The configured vibration signal is delivered from a signal source to the VCCS, where it is converted into a current signal. This current signal is then supplied to the two series-connected voice coil motors. The voice coil motors are mounted symmetrically on both sides of the vibration isolation platform. The constraint structure ensures that the isolation platform only undergoes vertical translation, with all other degrees of freedom being constrained [18]. The atom interferometer, mounted on a bracket, is positioned directly above the vibration source. The retroreflector is positioned above a seismometer, which is used to mark the applied vibration signal. The seismometer is centrally located on the top plate of the passive vibration isolation platform. The g at the site and background noise level were determined through half an hour of atom interferometer measurements conducted without vibration input. The vibration signal with a characteristic frequency of 0.05 Hz was transmitted from the signal source to voice coil motors. The voice coil motors then drove the top plate of the vibration isolation platform to generate a vibration at 0.05 Hz. Vibration measurements were conducted using the atom interferometer at a sampling rate of 1 Hz over a 30-min period. The mirror’s vibration was determined by subtracting the gravitational acceleration from the initial measurement results.

**Figure 3:**
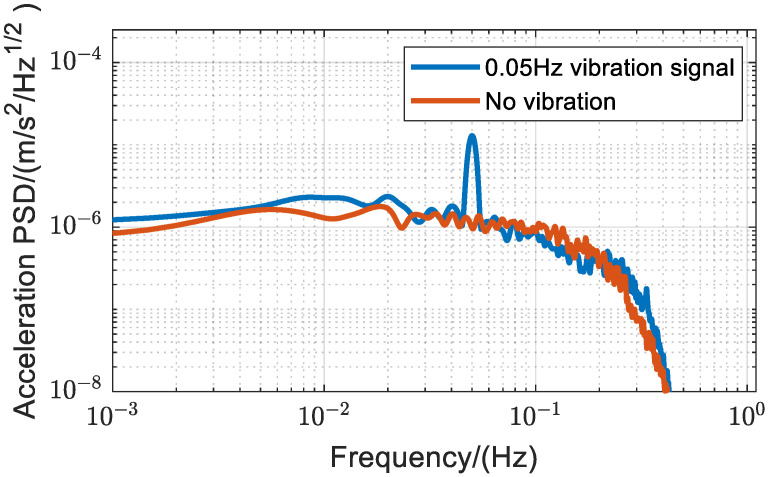
PSD of vibration measured by the atom interferometer. The measurement results for a 0.05 Hz vibration signal over 30 min.

The experimental results are shown in Figure 3. The red curve represents the PSD of vibration acceleration measured over 30 min without added vibrations. For frequencies below 0.01 Hz, PSD exhibits a near-flat profile with a slight non-zero slope, consistent with the characteristics of Gaussian white noise. The intercept of the PSD curve corresponds to the system’s best achievable sensitivity of 1.1 $\mu m/s^{2}/\sqrt{\mathrm{Hz}}$, representing the minimum detectable noise floor of the gravimeter. Compared to Figure 1b, the red curve reveals discrepancies in the 0.1–0.5 Hz frequency range. This arises because during actual measurements, a Proportional-Integral-Derivative (PID) loop is employed to dynamically adjust the frequency chirp α in actual vibration measurements. The PID loop in the system locks the frequency chirp with a bandwidth of approximately 0.125 Hz. Figure 3 validates these results [35]. The blue curve shows the PSD obtained after 30 min of measuring vibrations, including a 0.05 Hz sinusoidal signal. Comparing the PSD without vibrational input reveals a distinct peak at 0.05 Hz, while other regions are essentially unchanged.

**Figure 4:**
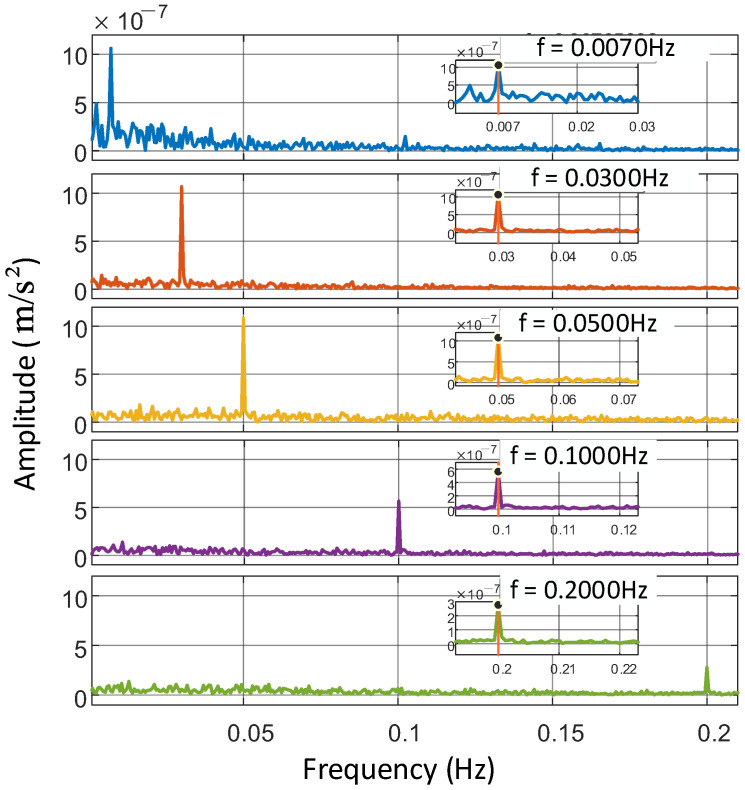
Five sets of vibration spectra measured by the atom interferometer across different frequencies. The frequencies span from 0.007 Hz to 0.2 Hz, with measurements conducted at a consistent amplitude over 30 min. The center is a magnified view of the peak frequency range in the spectrogram.

To further validate the bandwidth of the vibration measurement system, we tested its response to signals at five distinct frequencies: 0.2 Hz, 0.1 Hz, 0.05 Hz, 0.03 Hz, and 0.007 Hz. For consistency, the amplitude of the vibration signals was held constant across all frequencies. This was achieved by calibrating the output of the signal source to maintain a fixed amplitude while varying the frequency. The measurement results, shown in Figure 4, demonstrate the system’s performance across the tested bandwidth. The curves in Figure 4 represent spectrograms obtained from measurements at different frequencies. From the enlarged part of Figure 4, the measured signal frequency closely matches the input signal’s frequency. Given a sampling rate of 1 Hz and a total measurement duration of 30 min, the system’s frequency resolution is determined by the inverse of the measurement time, approximately $5.56\times 10^{-4}$ Hz. At 0.1 Hz and 0.2 Hz, the amplitude of the measured vibration signal decreases significantly. This is consistent with the PSD curve’s decrease above 0.1 Hz in Figure 3. A slight increase in the signal’s noise floor around 0.007 Hz is attributed to output vibration jitter caused by limitations in the voice coil motor’s performance. Theoretically, the system’s frequency response extends down to DC. However, to generate a vibration acceleration a of constant amplitude, the displacement produced by the vibration generator must follow an inverse proportionality with the square of its frequency. This implies that lower frequencies demand significantly larger mechanical displacements to maintain stable acceleration levels. The vibration source system used in this study has a maximum displacement capacity of 24.9 mm. To ensure undistorted output vibration from the vibration source system and maintain detectability by the atom interferometer, the lowest feasible vibration frequency is set to 0.007 Hz.

We verify the accuracy of this amplitude measurement method. A critical consideration is ensuring that the input vibration signal does not exceed the range of the interferometer during measurement. As derived in (6), at *T* = 105 ms, the acceleration should not change by more than 167.6 $\mu m/s^{2}$ within each period. To measure higher vibration accelerations, the interferometer’s range can be increased by decreasing *T*, though this results in reduced measurement accuracy.

**Figure 5:**
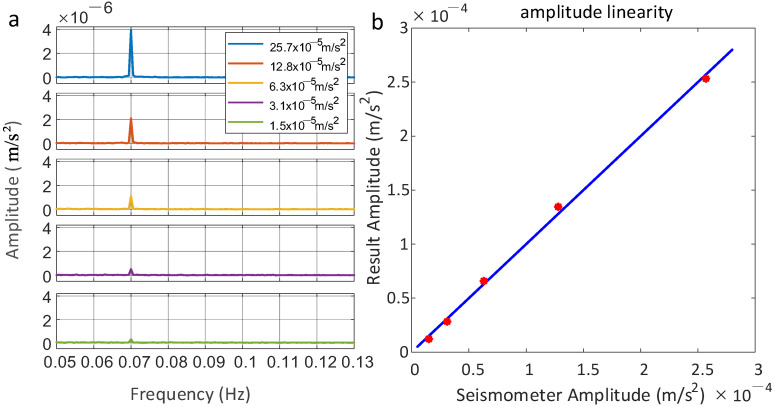
Results of vibration signals with varying amplitudes measured by the atom interferometer are presented. (**a**) Spectrograms illustrate the continuous measurement of vibrations at varying amplitudes over a 30-min period. (**b**) The results of linear regression fitting applied to the measurements are also presented.

When the frequency of the input signal to the voice coil motor is 0.07 Hz and the amplitude exceeds 80 mV, the impact of the vibration signal surpasses the range with a T of 105 ms. Accordingly, the value of T was reduced to 50 ms. The amplitudes for the five 30-min vibration measurement experiments were set at 10 mV, 20 mV, 40 mV, 80 mV, and 160 mV. The corresponding acceleration values measured by the seismometer were 15 $\mu m/s^{2}$, 31 $\mu m/s^{2}$, 63 $\mu m/s^{2}$, 128 $\mu m/s^{2}$, and 257 $\mu m/s^{2}$. The experimental results are depicted in Figure 5, with the spectral curves of the five groups of signal measurements represented in Figure 5a. Notably, as the amplitude of the input voltage signal increases linearly, the measured amplitude at a frequency of 0.07 Hz also exhibits a linear increase. The measurement results exhibit a fixed multiplicative difference compared to the seismometer’s calibration results, which can be attributed to the spatial difference between the sensitive mass inside the seismometer and the mirror used by the interferometer to detect vibrations. It can be observed from the system modeling that this discrepancy can be approximated as a linear relationship [29]. By calculating the corresponding transfer function and incorporating the vibration measurements from the atom interferometer, the vibration results from the seismometer at the corresponding locations can be calculated. The acceleration amplitudes measured by the seismometer are plotted on the x-axis, while the corresponding processed amplitudes from the atom interferometer are plotted on the y-axis in Figure 5b. The results show a strong agreement between the two measurement systems. For a 0.07 Hz vibration input with an amplitude of 257 μm/s^2^, the method demonstrated a measurement error of ±1.4%. The linear regression slope of 0.998 ± 0.026 in Figure 5b closely approximates the ideal unity slope, confirming excellent linearity and validating the atom interferometer’s accuracy against the seismometer reference.

**Figure 6:**
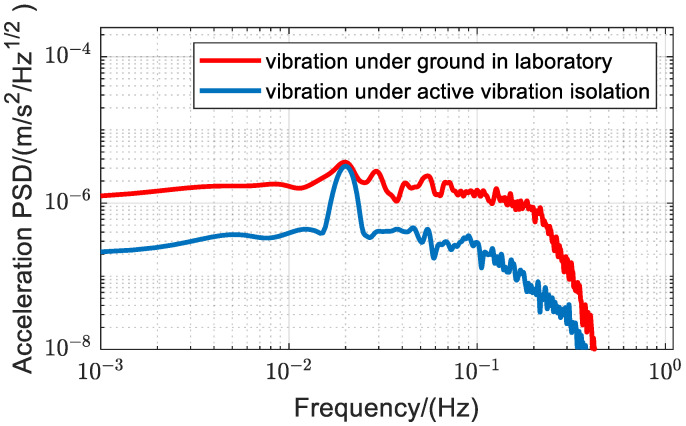
PSD of vibration measured by the atom interferometer. Under active isolation conditions, the baseline noise of the vibration measurement is approximately one order of magnitude lower compared to ground conditions. The red curve represents the power spectral density of the vibration signal applied by the voice coil motor on the passive isolation platform. The blue curve represents the power spectral density of the vibration signal applied by the speaker on the active isolation platform.

The measurement noise of vibration by this method is mainly due to the aliasing effect and phase noise of gravity measurement. We incorporated an active vibration isolation device capable of attenuating vibration signal amplitudes in the 0.1 Hz to 40 Hz range by more than −10 dB [6]. We first input a 0.02 Hz vibration signal from the source without active vibration isolation and measured vibrations using an atom interferometer over 30 min. The active vibration isolation system was then added. Here, the voice coil motors serve as actuators in the whole active vibration isolation system. We placed a commercial speaker serving as an independent vibration source near the vibration isolator. The speaker, also driven by the same signal source, produced 0.02 Hz vibrations. Measurements were made with an atom interferometer for 30 min while the active vibration isolation system was operational to allow for comparison. The results are shown in Figure 6. The red curve represents the PSD without vibration isolation, and the blue curve represents the PSD with vibration isolation, indicating a baseline noise reduction when vibration isolation is employed. An analysis of the PSD curve reveals that the sensitivity of this method can be improved to 0.2 $\mu m/s^{2}/\sqrt{\mathrm{Hz}}$ following the incorporation of active vibration isolation.

## 4. Conclusions

In conclusion, we demonstrate the feasibility of measuring low-frequency vibrations using atom interferometry. Although current experimental conditions limit the generation of vibration signals at lower frequencies, it is still possible to measure vibrations down to 0.007 Hz. It demonstrated that vibrations can indeed be measured down to 0.007 Hz with an optimal sensitivity of 1.1 $\mu m/s^{2}/\sqrt{\mathrm{Hz}}$, and this can be further enhanced through the combination with active vibration isolation, which improves accuracy by an additional order of magnitude. The optimal sensitivity can be improved to 0.2 $\mu m/s^{2}/\sqrt{\mathrm{Hz}}$. This method is capable of measuring ultra-low-frequency signals with high absolute sensitivity. With the continued development of atom interferometry and the further suppression of aliasing effects, as demonstrated in Table 1, its sensitivity is expected to reach the $1*10^{-8}\;m/s^{2}/\sqrt{\mathrm{Hz}}$ level in the future. This technique holds promise for applications in geological exploration, low-frequency shaker calibration, and related fields.

## Data Availability

The data in this study are available from the authors upon reasonable request.
